# Transfer learning for medical image classification: a literature review

**DOI:** 10.1186/s12880-022-00793-7

**Published:** 2022-04-13

**Authors:** Hee E. Kim, Alejandro Cosa-Linan, Nandhini Santhanam, Mahboubeh Jannesari, Mate E. Maros, Thomas Ganslandt

**Affiliations:** 1 Department of Biomedical Informatics at the Center for Preventive Medicine and Digital Health (CPD-BW), Medical Faculty Mannheim, Heidelberg University, Theodor-Kutzer-Ufer 1-3, 68167 Mannheim, Germany; 2 Chair of Medical Informatics, Friedrich-Alexander-Universität Erlangen-Nürnberg, Wetterkreuz 15, 91058 Erlangen, Germany

**Keywords:** Deep learning, Transfer learning, Fine-tuning, Convolutional neural network, Medical image analysis

## Abstract

**Background:**

Transfer learning (TL) with convolutional neural networks aims to improve performances on a new task by leveraging the knowledge of similar tasks learned in advance. It has made a major contribution to medical image analysis as it overcomes the data scarcity problem as well as it saves time and hardware resources. However, transfer learning has been arbitrarily configured in the majority of studies. This review paper attempts to provide guidance for selecting a model and TL approaches for the medical image classification task.

**Methods:**

425 peer-reviewed articles were retrieved from two databases, PubMed and Web of Science, published in English, up until December 31, 2020. Articles were assessed by two independent reviewers, with the aid of a third reviewer in the case of discrepancies. We followed the PRISMA guidelines for the paper selection and 121 studies were regarded as eligible for the scope of this review. We investigated articles focused on selecting backbone models and TL approaches including feature extractor, feature extractor hybrid, fine-tuning and fine-tuning from scratch.

**Results:**

The majority of studies (n = 57) empirically evaluated multiple models followed by deep models (n = 33) and shallow (n = 24) models. Inception, one of the deep models, was the most employed in literature (n = 26). With respect to the TL, the majority of studies (n = 46) empirically benchmarked multiple approaches to identify the optimal configuration. The rest of the studies applied only a single approach for which feature extractor (n = 38) and fine-tuning from scratch (n = 27) were the two most favored approaches. Only a few studies applied feature extractor hybrid (n = 7) and fine-tuning (n = 3) with pretrained models.

**Conclusion:**

The investigated studies demonstrated the efficacy of transfer learning despite the data scarcity. We encourage data scientists and practitioners to use deep models (e.g. ResNet or Inception) as feature extractors, which can save computational costs and time without degrading the predictive power.

**Supplementary Information:**

The online version contains supplementary material available at 10.1186/s12880-022-00793-7.

## Introduction

Medical image analysis is a robust subject of research, with millions of studies having been published in the last decades. Some recent examples include computer-aided tissue detection in whole slide images (WSI) and the diagnosis of COVID-19 pneumonia from chest images. Traditionally, sophisticated image feature extraction or discriminant handcrafted features (e.g. histograms of oriented gradients (HOG) features [1] or local binary pattern (LBP) features [2]) have dominated the field of image analysis, but the recent emergence of deep learning (DL) algorithms has inaugurated a shift towards non-handcrafted engineering, permitting automated image analysis. In particular, convolutional neural networks (CNN) have become the workhorse DL algorithm for image analysis. In recent data challenges for medical image analysis, all of the top-ranked teams utilized CNN. For instance, the top-ten ranked solutions, excepting one team, had utilized CNN in the CAMELYON17 challenge for automated detection and classification of breast cancer metastases in whole slide images [3]. It has also been demonstrated that the features extracted from DL surpassed that of the handcrafted methods by Shi et al. [4].

However, DL algorithms including CNN require—under preferable circumstances—a large amount of data for training; hence follows the data scarcity problem. Particularly, the limited size of medical cohorts and the cost of expert-annotated data sets are some well-known challenges. Many research endeavors have tried to overcome this problem with transfer learning (TL) or domain adaptation [5] techniques. These aim to achieve high performance on target tasks by leveraging knowledge learned from source tasks. A pioneering review paper of TL was contributed by Pan and Yang [6] in 2010, and they classified TL techniques from a labeling aspect, while Weiss et al. [7] summarized TL studies based on homogeneous and heterogeneous approaches. Most recently in 2020, Zhuang et al. [8] reviewed more than forty representative TL approaches from the perspectives of data and models. Unsupervised TL is an emerging subject and has recently received increasing attention from researchers. Wilson and Cook [9] surveyed a large number of articles of unsupervised deep domain adaptation. Most recently, generative adversarial networks (GANs)-based frameworks [10–12] gained momentum, a particularly promising approach is DANN [13]. Furthermore, multiple kernel active learning [14] and collaborative unsupervised methods [15] have also been utilized for unsupervised TL.

Some studies conducted a comprehensive review focused primarily on DL in the medical domain. Litjens et al. [16] reviewed DL for medical image analysis by summarizing over 300 articles, while Chowdhury et al. [17] reviewed the state-of-the-art research on self-supervised learning in medicine. On the other hand, others surveyed articles focusing on TL with a specific case study such as microorganism counting [18], cervical cytopathology [19]**,** neuroimaging biomarkers of Alzheimer's disease [20] and magnetic resonance brain imaging in general [21].

In this paper, we aimed to conduct a survey on TL with pretrained CNN models for medical image analysis across use cases, data subjects and data modalities. Our major contributions are as follows:

(i) An overview of contributions to the various case studies is presented;
(ii) Actionable recommendations on how to leverage TL for medical image classification are provided;
(iii) Publicly available medical datasets are compiled with URL as a supplementary material.

**Fig. 1:**
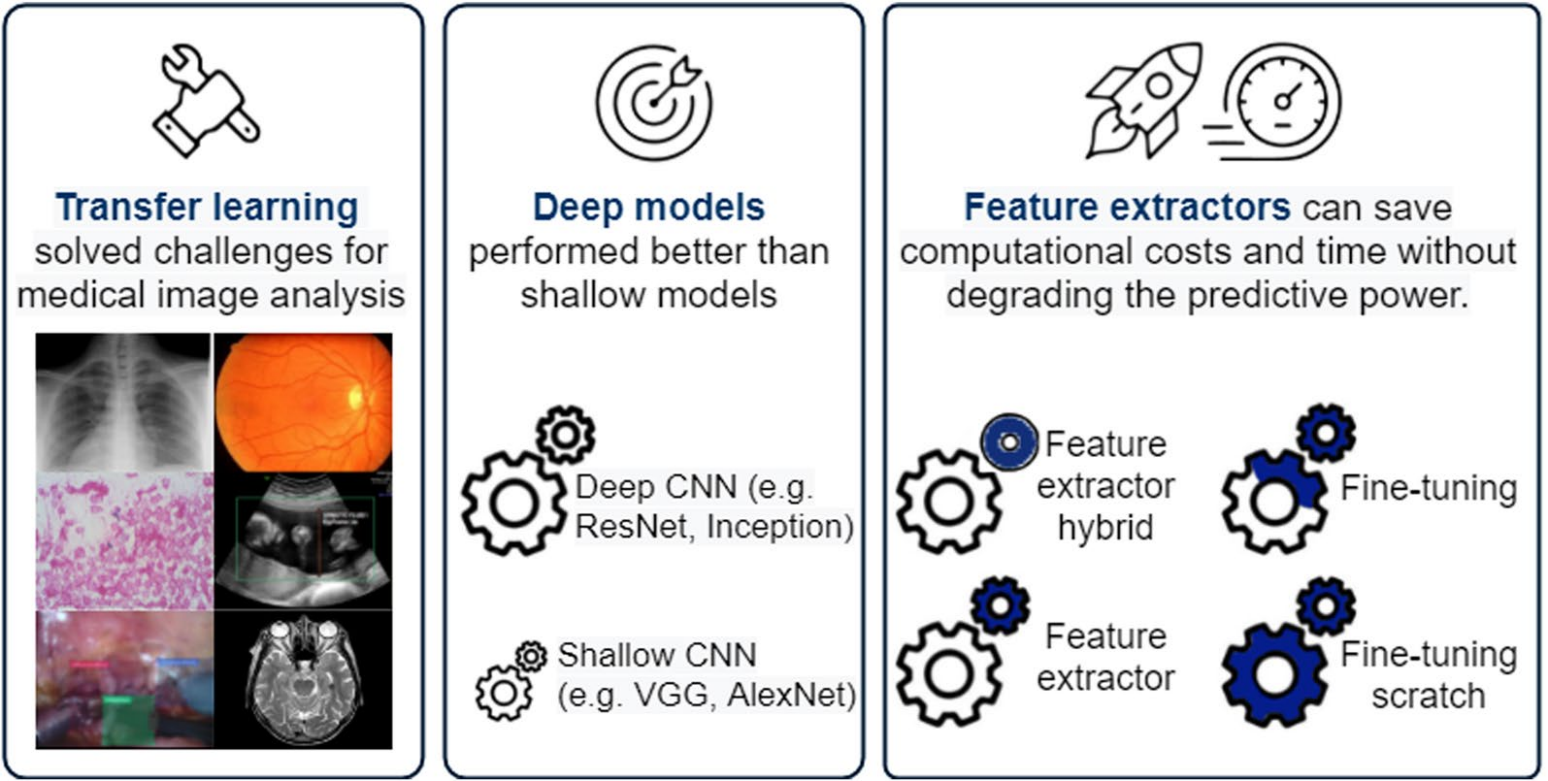
Visual abstract summarizing the scope of our study

The rest of this paper is organized as follows. Section 2 covers the background knowledge and the most common notations used in the following sections. In Sect. 3, we describe the protocol for the literature selection. In Sect. 4, the results obtained are analyzed and compared. Critical discussions are presented in Sect. 5. Finally, we end with a conclusion and the lessons learned in Sect. 6. Figure 1 is the main diagram which presents the whole manuscript.

## Background

### Transfer learning

Transfer learning (TL) stems from cognitive research, which uses the idea, that knowledge is transferred across related tasks to improve performances on a new task. It is well-known that humans are able to solve similar tasks by leveraging previous knowledge. The formal definition of TL is defined by Pan and Yang with notions of domains and tasks. “A domain consists of a feature space $\mathcal{X}$ and marginal probability distribution$P(X)$, where$X=\{{x}_{1}, ..., {x}_{n }\}\in \mathcal{X}$. Given a specific domain denoted by$D=\left\{\mathcal{X}, P(X)\right\}$, a task is denoted by $\mathcal{T}=$ $\left\{\mathcal{Y}, f(\cdot )\right\}$ where $\mathcal{Y}$ is a label space and $f(\cdot )$ is an objective predictive function. A task is learned from the pair $\{{x}_{i}, {y}_{i}\}$ where ${x}_{i}\in \mathcal{X}$ and${y}_{i}\in \mathcal{Y}$. Given a source domain ${{\varvec{D}}}_{{\varvec{S}}}$ and learning task${{\varvec{T}}}_{{\varvec{S}}}$, a target domain ${{\varvec{D}}}_{{\varvec{T}}}$ and learning task${{\varvec{T}}}_{{\varvec{T}}}$, transfer learning aims to improve the learning of the target predictive function ${f}_{T}$(·) in ${D}_{T}$ by using the knowledge in ${{\varvec{D}}}_{{\varvec{S}}}$ and${{\varvec{T}}}_{{\varvec{S}}}$” [6].

Analogously, one can learn how to drive a motorbike ${{\varvec{T}}}_{{\varvec{T}}}$ (transferred task) based on one’s cycling skill ${{\varvec{T}}}_{{\varvec{s}}}$ (source task) where driving two-wheel vehicles is regarded as the same domain ${{\varvec{D}}}_{{\varvec{S}}}$ $=$ ${{\varvec{D}}}_{{\varvec{T}}}$. This does not mean that one will not learn how to drive a motorbike without riding a bike, but it takes less effort to practice driving the motorbike by adapting one’s cycling skills. Similarly, learning the parameters of a network from scratch will require larger annotated datasets and a longer training time to achieve an acceptable performance.

### Convolutional neural networks using imageNet

Convolutional neural networks (CNN) are a special type of deep learning that processes grid-like topology data such as image data. Unlike the standard neural network consisting of fully connected layers only, CNN consists of at least one convolutional layer. Several pretrained CNN models are publicly accessible online with downloadable parameters. They were pretrained with millions of natural images on the ImageNet dataset (ImageNet large scale visual recognition challenge; ILSVRC) [22].

In this paper, CNN models are denoted as backbone models. Table 1 summarizes the five most popular models in chronological order from top to bottom. LeNet [23] and AlexNet [24] are the first generations of CNN models developed in 1998 and 2012 respectively. Both are relatively shallow compared to other models that are developed recently. After AlexNet won the ImageNet large scale visual recognition challenge (ILSVRC) in 2012, designing novel networks became an emerging topic among researchers. VGG [25], also referred to as OxfordNet, is recognized as the first deep model, while GoogLeNet [26], also known as Inception1, set the new state of the art in the ILSVRC 2014. Inception introduced the novel block concept that employs a set of filters with different sizes, and its deep networks were constructed by concatenating the multiple outputs. However, in the architecture of very deep networks, the parameters of the earlier layers are poorly updated during training because they are too far from the output layer. This problem is known as the vanishing gradient problem which was successfully addressed by ResNet [27] by introducing residual blocks with skip connections between layers.

**Table 1:** Overview of five backbone models

Model type	Model	Released year	Parameters (all)	Parameters (FE only)	Trainable layers (FE + FC layers)	Dataset
Shallow and linear	LeNet5	1998	60,000	1,716	4 (2 + 2)	MNIST
	AlexNet	2012	62.3 M	3.7 M	8 (5 + 3)	ImageNet
	VGG16	2014	134.2 M	14.7 M	16 (13 + 3)	
Deep	GoogLeNet	2014	5.3 M	5.3 M	22 (21 + 1)	
	ResNet50	2015	25.6 M	23.5 M	51 (50 + 1)	

FE: feature extraction, FC: fully connected layers; MNIST database: Modified National Institute of Standards and Technology database of handwritten digits with 60,000 training and 10,000 test images, ImageNet database: organized according to the WordNet hierarchy with over 14 million hand-annotated images for visual object recognition research

The number of parameters of one filter is calculated by (a * b * c) + 1, where a * b is the filter dimension, c is the number of filters in the previous layer and added 1 is the bias. The total number of parameters is the summation of the parameters of each filter. In the classifier head, all models use the Softmax function except LeNet-5, which utilizes the hyperbolic tangent function. The Softmax function fits well with the classification problem because it can convert feature vectors to the probability distribution for each class candidate.

### Transfer learning with convolutional neural networks

TL with CNN is the idea that knowledge can be transferred at the parametric level. Well-trained CNN models utilize the parameters of the convolutional layers for a new task in the medical domain. Specifically, in TL with CNN for medical image classification, a medical image classification (target task) can be learned by leveraging the generic features learned from the natural image classification (source task) where labels are available in both domains. For simplicity, the terminology of TL in the remainder of the paper refers to homogeneous TL (i.e. both domains are image analysis) with pretrained CNN models using ImageNet data for medical image classification in a supervisory manner.

**Fig. 2:**
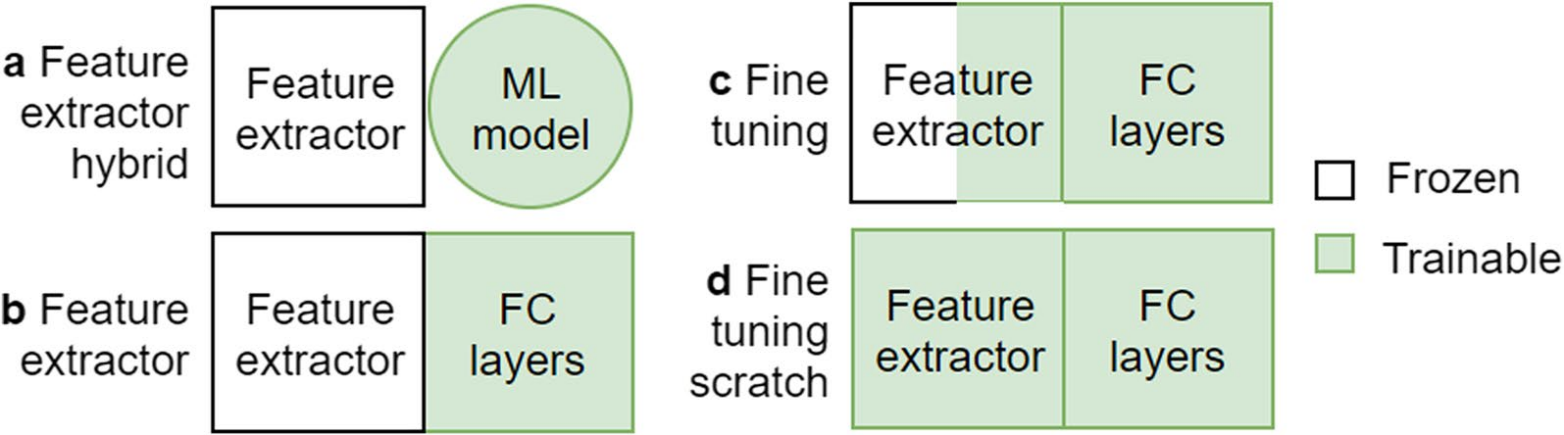
Four types of transfer learning approach. The last classifier block needs to be replaced by a thinner layer or trained from scratch (ML: Machine learning; FC: Fully connected layers)

Roughly, there are two TL approaches to leveraging CNN models: either feature extractor or fine-tuning. The feature extractor approach freezes the convolutional layers, whereas the fine-tuning approach updates parameters during model fitting. Each can be further divided into two subcategories; hence, four TL approaches are defined and surveyed in this paper. They are intuitively visualized in Fig. 2. Feature extractor hybrid (Fig. 2a) discards the FC layers and attaches a machine learning algorithm such as SVM or Random Forest classifier into the feature extractor, whereas the skeleton of the given networks remains the same in the other types (Fig. 2b-d). Fine-tuning from scratch is the most time-intensive approach because it updates the entire ensemble of parameters during the training process.

## Methods

Publications were retrieved from two peer-reviewed databases (PubMed database on January 2, 2021, and Web of Science database on January 22, 2021). Papers were selected based on the following four conditions: (1) convolutional or CNN should appear in the title or abstract; (2) image data analysis should be considered; (3) “transfer learning” or “pretrained” should appear in the title or abstract; finally, (4) only experimental studies were considered. The time constraint is specified only for the latest date, which is December 31, 2020. The exact search strings used for these two databases are denoted in Appendix A. Duplicates were merged before screening assessment. The first author screened the title, abstract and methods in order to exclude studies proposing a novel CNN model. Typically, this type of study stacked up multiple CNN models or concatenated CNN models and handcrafted features, and then compared its efficacy with other CNN models. Non-classification tasks, and those publications which fell outside the aforementioned date range, were also excluded. For the eligibility assessment, full texts were examined by two researchers. A third, independent researcher was involved in decision-making in the case of discrepancy between the two researchers.

### Methodology analysis

Eight properties of 121 research articles were surveyed, investigated, compared and summarized in this paper. Five are quantitative properties and three are qualitative properties. They are specified as follows: (1) Off-the-shelf CNN model type (AlexNet, CaffeNet, Inception1, Inception2, Inception3, Inception4, Inception-Resnet, LeNet, MobileNet, ResNet, VGG16, VGG19, DenseNet, Xception, many or else); (2) Model performances (accuracy, AUC, sensitivity and specificity); (3) Transfer learning type (feature extractor, feature extractor hybrid, fine-tuning, fine-tuning or many); (4) Fine-tuning ratio; (5) Data modality (endoscopy, CT/CAT scan, mammographic, microscopy, MRI, OCT, PET, photography, sonography, SPECT, X-ray/radiography or many); (6) Data subject (abdominopelvic cavity, alimentary system, bones, cardiovascular system, endocrine glands, genital systems, joints, lymphoid system, muscles, nervous system, tissue specimen, respiratory system, sense organs, the integument, thoracic cavity, urinary system, many or else); (7) Data quantity; and (8) The number of classes. They fall into one of three categories, namely model, transfer learning or data.

## Results

**Fig. 3:**
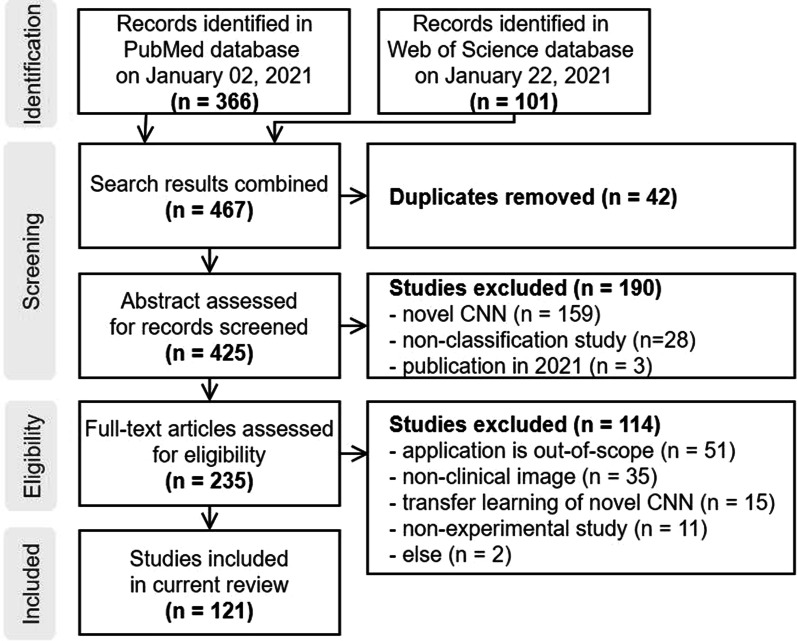
Flowchart of the literature search

Figure 3 shows the PRISMA flow diagram of paper selection. We initially retrieved 467 papers from PubMed and Web of Science. 42 duplicates were merged from two databases, and then 425 studies were assessed for screening. 189 studies were excluded during the screening phase, and then full texts of 236 studies were assessed for the next stage. 114 studies were disqualified from inclusion, resulting in 121 studies. These selected studies were further investigated and organized with respect to their backbone model and TL type. The data characteristics and model performance were also analyzed to gain insights regarding how to employ TL.

**Fig. 4:**
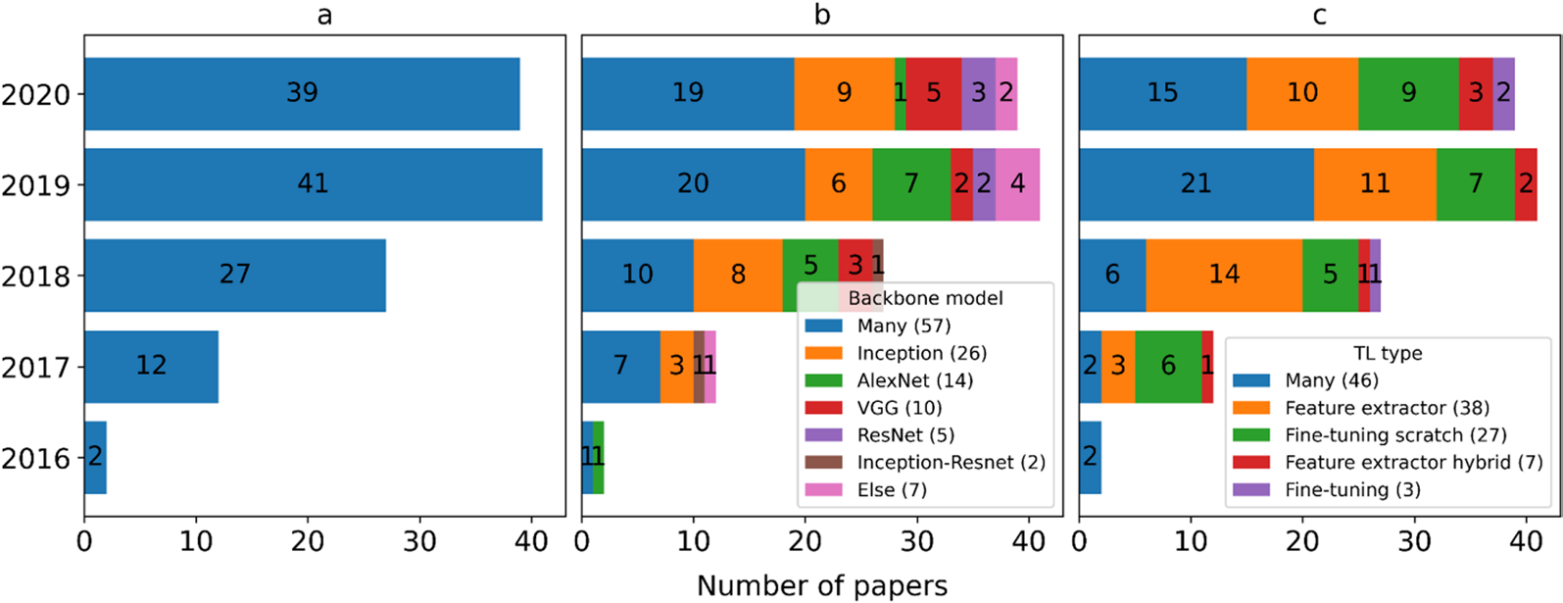
Studies of transfer learning in medical image classification over time (y-axis) with respect to **a** the number of publications, **b** applied backbone model and **c** transfer learning type

Figure 4a shows that studies of TL for medical image classification have emerged since 2016 with a 4-year delay after AlexNet [24] won the ImageNet Challenge in 2012. Since then the number of publications grew rapidly for consecutive years. Studies published in 2020 seem shrinking compared to the number of publications in 2019, because the process of indexing a publication may take anywhere from three to six months.

### Backbone model

The majority of the studies (n = 57) evaluated several backbone models empirically as depicted in Fig. 4b. For example, Rahaman and his colleagues [28] contributed an intensive benchmark study by evaluating fifteen models, namely: VGG16, VGG19, ResNet50, ResNet101, ResNet152, ResNet50V2, ResNet101V2, ResNet152V2, Inception3, InceptionResNet2, MobileNet1, DenseNet121, DenseNet169, DenseNet201 and XceptionNet. They concluded that VGG19 presented the highest accuracy of 89.3%. This result is exceptional because other studies reported that deeper models (e.g. Inception and ResNet) performed better than the shallow models (e.g. VGG and AlexNet). Five studies [29–33] compared Inception and VGG and reported that Inception performed better, and Ovalle-Magallanes et al. [34] also concluded that Inception3 outperformed compared to ResNet50 and VGG16. Finally, Talo et al. [35] reported that ResNet50 achieved the best classification accuracy compared to AlexNet, VGG16, ResNet18 and ResNet34.

Besides the benchmark studies, the most prevalent model was the Inception (n = 26) that consists of the least parameters shown in Table 1. AlexNet (n = 14) and VGG (n = 10) were the next commonly used models although they are shallower than ResNet (n = 5) and Inception-Resnet (n = 2). Finally, only a few studies (n = 7) used a specific model such as LeNet5, DenseNet, CheXNet, DarkNet, OverFeat or CaffeNet.

### Transfer learning

Similar to the backbone model, the majority of models (n = 46) evaluated numerous TL approaches, which are illustrated in Fig. 4c. Many researchers aimed to search for the optimal choice of TL approach. Typically, grid search was applied. Shin and his colleagues [36] extensively evaluated three components by varying three CNN models (CifarNet, AlexNet and GoogLeNet) with three TL approaches (feature extractor, fine-tuning from scratch with and without random initialization), and the fine-tuned GoogLeNet from scratch without random initialization was identified as the best performing model.

The most popular TL approach was feature extractor (n = 38) followed by fine-tuning from scratch (n = 27), feature extractor hybrid (n = 7) and fine-tuning (n = 3). Feature extractor takes the advantage of saving computational costs by a large degree compared to the others. Likewise, the feature extractor hybrid can profit from the same advantage by removing the FC layers and adding less expansive machine learning algorithms. This is particularly beneficial for CNN models with heavy FC layers like AlexNet and VGG. Fine-tuning from scratch was the second most popular approach despite it being the most resource-expensive type because it updates the entire model. Fine-tuning is less expensive compared to the fine-tuning from scratch as it partially updates the parameters of the convolutional layers. Additional file 2: Table 2 in Appendix B presents an overview of four TL approaches which were organized based on three dimensions: data modality, data subject and TL type.

### Data characteristics

**Fig. 5:**
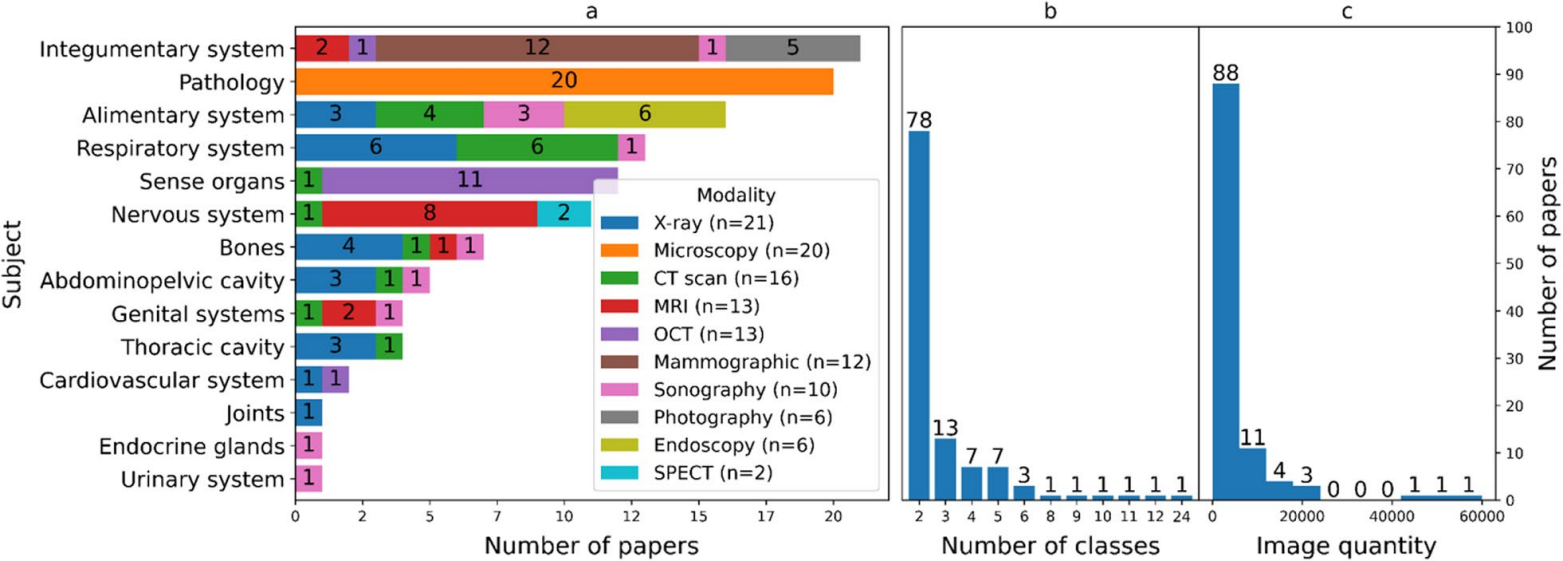
The overview of data characteristics of selected publications. **a** The correlation of anatomical body parts and imaging modalities. **b** The number of classes **c** The histogram of the quantity of medical image datasets

As the summary of data characteristics is depicted in Fig. 5, a variety of human anatomical regions has been studied. Most of the studied regions were breast cancer exams and skin cancer lesions. Likewise, a wide variety of imaging modalities contained a unique attribute of medical image analysis. For instance, computed tomography (CT) scans and magnetic resonance imaging (MRI) are capable of generating 3D image data, while digital microscopy can generate terabytes of whole slide image (WSI) of tissue specimens.

Figure 5b shows that the majority of studies consist of binary classes, while Fig. 5c shows that the majority of studies have fallen into the first bin which ranges from 0 to 600. Minor publications are not depicted in Fig. 5 for the following reasons: the experiment was conducted with multiple subjects (human body parts); multiple tasks; multiple databases; or the subject is non-human body images (e.g. surgical tools).

### Performance visualization

**Fig. 6:**
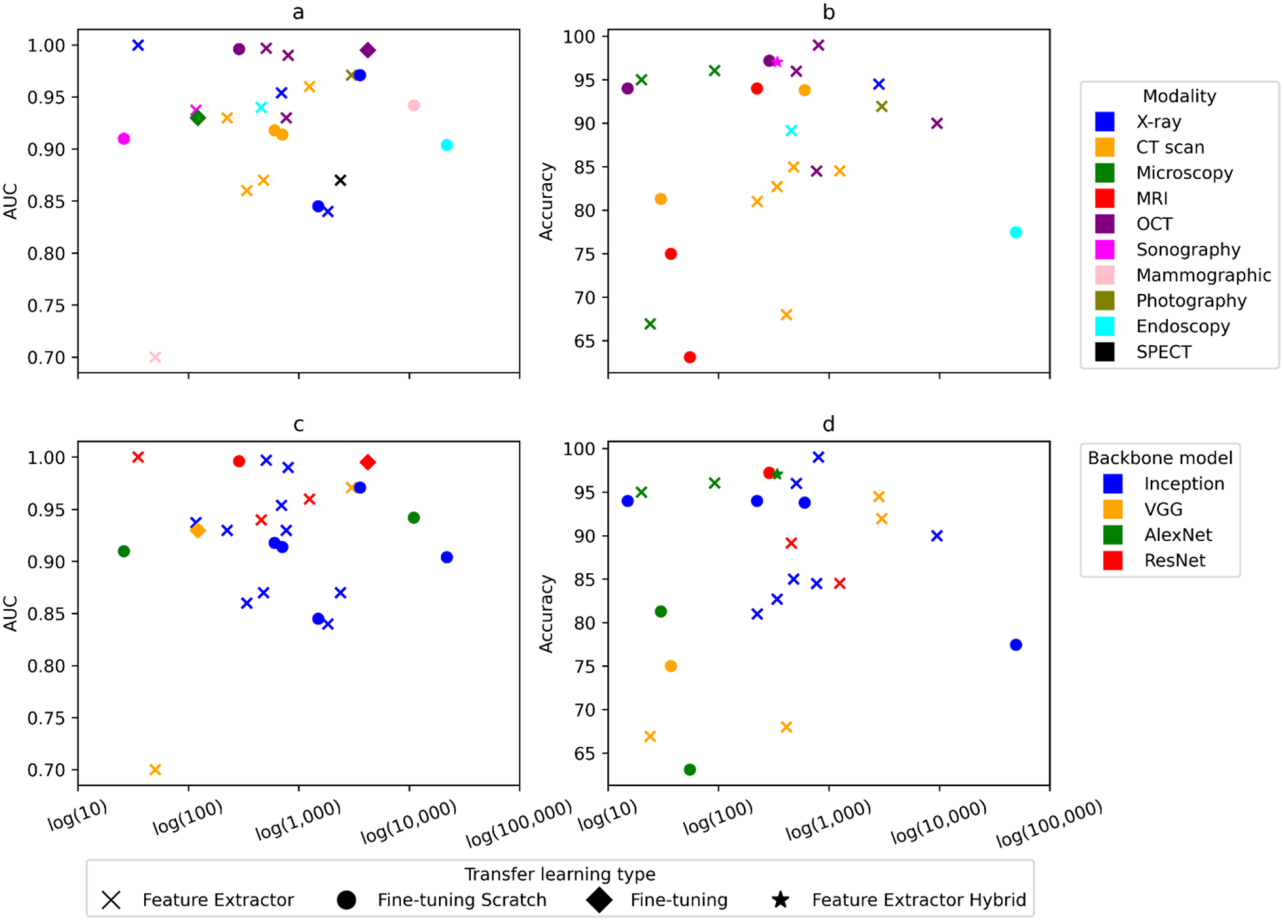
Scatter plots of model performance with data size, image modality, backbone model and transfer learning type. Color keys in **a** and **b** indicate the medical image modality, whereas color keys in **c** and **d** represent backbone models. Transfer learning types are in any of four marker shapes for all subfigures

Figure 6 shows scatter plots of model performance, TL type and two data characteristics: data size and image modality. The Y coordinates adhere to two metrics, namely area under the receiver operating characteristic curve (AUC) and accuracy. Eleven studies used both metrics, so they are displayed on both scatter plots. The X coordinate is the normalized data quantity, otherwise it is not fair to compare the classification performance with two classes versus ten classes. The data quantities of three modalities—CT, MRI and Microscopy—reflect the number of patients.

For the fair comparison, studies employed only a single model, TL type and image modality are depicted (n = 41). Benchmark studies were excluded; otherwise, one study would generate several overlapping data points and potentially lead to bias. The excluded studies are either with multiple models (n = 57), with multiple TL types (n = 14) or with minor models like LeNet (n = 9).

According to Spearman’s rank correlation analyses, there were no relevant associations observed between the size of the data set and performance metrics. Data size and AUC (Fig. 6a, c) showed no relevant correlation (r_sp_ = 0.05, p = 0.03). Similarly, only a weak positive trend (r_sp_ = 0.13, p = 0.17) could be detected between the size of the dataset and accuracy (Fig. 6b, d). There was also no association between other variables such as modality, TL type and backbone model. For instance, the data points of models, such as feature extractors that were fitted into optical coherence tomography (OCT) images (purple crosses, Fig. 6a, b) showed that larger data quantities did not necessarily guarantee better performance. Notably, data points in cross shapes (models as feature extractors) showed decent results even though only a few fully connected layers were being retrained.

## Discussion

In this survey of selected literature, we have summarized 121 research articles applying TL to medical image analysis and found that the most frequently used model was Inception. Inception is a deep model, nevertheless, it consists of the least parameters (Table 1) owing to the 1 × 1 filter [37]. This 1 × 1 filter acts as a fully connected layer in Inception and ResNet and it lowers the computational burden to a great degree [38]. To our surprise, AlexNet and VGG were the next popular models. At first glance, this result seemed counterintuitive because ResNet is a more powerful model with fewer parameters compared to AlexNet or VGG. For instance, ResNet50 achieved a top-5 error of 6.7% on ILSVRC, which was 2.6% lower than VGG16 with 5.2 times fewer parameters and 9.7% lower than AlexNet with 2.4 times fewer parameters [27]. However, this assumption is valid only if the model was fine-tuned from scratch. The number of parameters significantly drops when the model is utilized as a feature extractor as shown in Table 1. He et al. [39] performed an in-depth evaluation of the impact of various settings for refining the training of multiple backbone models, focusing primarily on the ResNet architecture. Another assumption was that AlexNet and VGG are easy to understand because the network morphology is linear and made up of stacked layers. This stands against more complex concepts such as skip connections, bottlenecks, convolutional blocks introduced in Inception or ResNet.

With respect to TL approaches, the majority of studies empirically tested as many possible combinations of CNN models with as many as possible TL approaches. Compared to previously suggested best practices [40], some studies determined fine-tuning arbitrarily and ambiguously. For instance, [41] froze all layers except the last 12 layers without justification, while [42, 43] did not clearly describe the fine-tuning configuration. Lee et al. [44] partitioned VGG16/19 into 5 blocks, unfroze blocks sequentially and identified the model fine-tuned with two blocks that achieved the highest performance. Similarly, fine-tuned CaffeNet by unfreezing each layer sequentially [45]. The best results were obtained by the model with one retrained layer for the detection task and with two retrained layers for the classification task.

Fine-tuning from scratch (n = 27) was a prevalent TL approach in the literature, however, we recommend using this approach carefully for two reasons: firstly, it does not improve the model performance as shown in Fig. 6 and secondly, it is the computationally most expensive choice because it updates large gradients for entire layers. Therefore, we encourage one to begin with the feature extractor approach, then incrementally fine-tune the convolutional layers. We recommend updating all layers (fine-tuning from scratch), if the feature extractor does not reflect the characteristics of the new medical images.

There was no consensus among studies concerning the global optimum configuration for fine-tuning. [46] concluded that fine-tuning the last fully connected layers of Inception3, ResNet50, and DenseNet121 outperformed fine-tuning from scratch in all cases. On the other hand, Yu et al. [47] found that retraining from scratch of DenseNet201 achieved the highest diagnostic accuracy. We speculate that one of the causes is the variety of data subjects and imaging modalities addressed in Sect. 4.3. Hence, investigating the medical data characteristics (e.g. anatomical sites, imaging modalities, data size, label size and more) and TL with CNN models would be interesting to investigate, yet it is understudied in the current literature. Morid et al. [48] stated that deep CNN models may be more effective for the following image modalities: X-ray, endoscopic and ultrasound images, while shallow CNN models may be optimal for processing these image modalities: OCT and photography for skin lesions and fundus. Nonetheless, more research is needed to further confirm these hypotheses.

TL with random initialization often appeared in the literature [49–52]. These studies used the architecture of CNN models only and initialized the training with random weights. One could argue that there is no transfer of knowledge if the entire weights and biases are initialized, but this is still considered as TL in the literature.

It is also worth noting that only a few studies [53, 54] employed native 3D-CNN. Both studies reported that 3D-CNN outperformed 2D-CNN and 2.5-CNN models, however, Zhang et al. [53] set the number of the frames to 16 and Xiong et al. [54] reduced the resolution up to 21*21*21 voxels due to the limitation of computer resources. The majority of the studies constructed 2D-CNN or 2.5D-CNN from 3D inputs. In order to reduce the processing burden, only a sample of image slices from 3D inputs was taken. We expect that the number of studies employing 3D models will increase in the future as high-performance DL is an emerging research topic.

We confirmed (Fig. 5c) that only a limited amount of data was available in most studies for medical image analysis. Many studies took advantage of using publicly accessible medical datasets from grand challenges (https://grand-challenge.org/challenges). This is a particularly beneficial scientific practice because novel solutions are shared online allowing for better reproducibility. We summarized 78 publicly available medical datasets in Additional file 3: Suppl. Table 3 (Appendix C), which were organized based on the following five attributes: data modality, anatomical part/region, task type, data name, published year and the link.

Although most evaluated papers included only brief information about their hardware setup, no details were provided about training or test time performance. As most medical data sets are small, usually consumer-grade GPUs in custom workstations or seldom server-grade cards (P100 or V100) were sufficient for TL. Previous survey studies have investigated how DL can be optimized and sped up on GPUs [55] or by using specifically designed hardware accelerators like field-programmable gate arrays (FPGA) for neural network inference [56]. We could not investigate these aspects of efficient TL because execution time was rarely reported in the surveyed literature.

This study is limited to surveying only TL for medical image classification. However, many interesting task-oriented TL studies were published in the past few years, with a particular focus on object detection and image segmentation [57], as reflected by the amount of public data sets (see also Additional file 3: Appendix C., Table 3). We only investigated off-the-shelf CNN models pretrained on ImageNet and intentionally left out custom CNN architectures, although these can potentially outperform TL-based models on certain tasks [58, 59]. Also, we did not evaluate aspects of potential model improvements leveraged by the differences of the source- and the target domain of the training data used for TL [60]. Similarly, we did not evaluate vision transformers (ViT) [61], which are emerging for image data analysis. For instance, Liu et al. [62] compared 22 backbone models and four ViT models and concluded that one of the ViT models exhibited the highest accuracy trained on cropped cytopathology cell images. Recently, Chen et al. [63] proposed a novel architecture that is a parallel design of MobileNet and ViT, in view of achieving not only more efficient computation but also better model performance.

## Conclusion

We aimed to provide actionable insights to the readers and ML practitioners, on how to select backbone CNN models and tune them properly with consideration of medical data characteristics. While we encourage readers to methodically search for the optimal choice of model and TL setup, it is a good starting point to employ deep CNN models (preferably ResNet or Inception) as feature extractors. We recommend updating only the last fully connected layers of the chosen model on the medical image dataset. In case the model performance needs to be refined, the model should be fine-tuned by incrementally unfreezing convolutional layers from top to bottom layers with a low learning rate. Following these basic steps can save computational costs and time without degrading the predictive power. Finally, publicly accessible medical image datasets were compiled in a structured table describing the modality, anatomical region, task type and publication year as well as the URL for accession.

## Supplementary Information

**Additional file 1.**  Search terms.

**Additional file 2.**  Summary table of studies.

**Additional file 3.** Summary table of public medical datasets.

## Data Availability

The dataset analyzed in this study are shown in Appendix B. In-depth information is available on reasonable request from the corresponding author (HeeEun.Kim@medma.uni-heidelberg.de).

## Abbreviations

AUC: Area under the receiver operating characteristic curve
CT: Computed tomography
CNN: Convolutional neural networks
DL: Deep learning
FC: Fully connected
FPGA: Field-programmable gate arrays
GPU: Graphics processing unit
HOG: Histograms of oriented gradients
ILSVRC: ImageNet large scale visual recognition challenge
LBP: Local binary pattern
MRI: Magnetic resonance imaging
OCT: Optical coherence tomography
TL: Transfer learning
TPU: Tensor processing unit
ViT: Vision transformer
WSI: Whole slide image
