# Supplementary material for: Transfer learning for medical image classification: a literature review
Source: BMC Med Imaging. 2022 Apr 13;22:69. doi: 10.1186/s12880-022-00793-7 (PMC9007400; doi:10.1186/s12880-022-00793-7)
Supplement: Supplementary file 1 — Additional file 1. Search terms. [file 12880_2022_793_MOESM1_ESM.docx]

# Appendix A. Search terms

The search terms used for PubMed were as follows: *("Convolutional neural network*"[Title/Abstract] OR "CNN*"[Title/Abstract]) AND ("image processing, computer-assisted"[MeSH Terms] OR "Diagnostic Imaging"[MeSH Terms] OR "medical imag*"[Title/Abstract] OR "clinical imag*"[Title/Abstract] OR "biomedical imag*") AND ("transfer learning"[Title/Abstract] OR "pre-trained"[Title/Abstract] OR "pretrained"[Title/Abstract]) NOT* *("Review"[Publication Type] OR "Letter"[Publication Type] OR "meta-analysis"[Publication Type] OR "Systematic Review"[Publication Type] OR "Systematic Review"[Publication Type])*

*The search string applied in Web of Science database was as follows: TS=("CNN" OR "convolutional") AND TS=("medical imag*" OR "clinical imag*" OR "biomedical imag*") AND TS=("transfer learning" OR "pre-trained" OR "pretrained") NOT TS=("novel" OR "propose")*
