# Supplementary material for: Transfer learning for medical image classification: a literature review
Source: BMC Med Imaging. 2022 Apr 13;22:69. doi: 10.1186/s12880-022-00793-7 (PMC9007400; doi:10.1186/s12880-022-00793-7)
Supplement: Supplementary file 2 — Additional file 2. Summary table of studies. [file 12880_2022_793_MOESM2_ESM.docx]

# Appendix B. Supplementary data

**Table 2** A summary table of studies that utilized transfer learning.

| **Modality** | **Subject** | **Transfer Learning** | **Reference** |
| --- | --- | --- | --- |
| CT scan | Abdominopelvic cavity | Feature extractor | [[64]](https://www.zotero.org/google-docs/?0ORLql) |
|  | Alimentary system | Feature extractor | [[65, 66]](https://www.zotero.org/google-docs/?YHSSc1) |
|  |  | Fine-tuning scratch | [[67, 68]](https://www.zotero.org/google-docs/?VH16yI) |
|  | Bones | Feature extractor | [[69]](https://www.zotero.org/google-docs/?MfnTeK) |
|  | Genital systems | Fine-tuning scratch | [[70]](https://www.zotero.org/google-docs/?sLGlOU) |
|  | Nervous system | Many | [[71]](https://www.zotero.org/google-docs/?ZaDc1t) |
|  | Respiratory system | Feature extractor | [[72]](https://www.zotero.org/google-docs/?pouwO3) |
|  |  | Feature extractor hybrid | [[73]](https://www.zotero.org/google-docs/?WL9SFR) |
|  |  | Fine-tuning scratch | [[74–76]](https://www.zotero.org/google-docs/?Q7bCfv) |
|  |  | Many | [[54, 77]](https://www.zotero.org/google-docs/?cqOhQl) |
|  | Sense organs | Feature extractor | [[78]](https://www.zotero.org/google-docs/?6Rt6g5) |
|  | Thoracic cavity | Feature extractor | [[79]](https://www.zotero.org/google-docs/?FqCtds) |
| Endoscopy | Alimentary system | Feature extractor | [[80, 81]](https://www.zotero.org/google-docs/?B7OE1w) |
|  |  | Fine-tuning scratch | [[82–84]](https://www.zotero.org/google-docs/?kmJ7og) |
|  |  | Many | [[85]](https://www.zotero.org/google-docs/?1ZGcVE) |
| Mammographic | Integumentary system | Feature extractor | [[4]](https://www.zotero.org/google-docs/?HLLwC7) |
|  |  | Feature extractor hybrid | [[86]](https://www.zotero.org/google-docs/?3cB6OX) |
|  |  | Fine-tuning scratch | [[33, 87]](https://www.zotero.org/google-docs/?bshftO) |
|  |  | Many | [[47, 53, 88–93]](https://www.zotero.org/google-docs/?bqWxii) |
| Microscopy | Tissues | Feature extractor | [[94–100]](https://www.zotero.org/google-docs/?4n3Rmf) |
|  |  | Fine-tuning | [[42]](https://www.zotero.org/google-docs/?ePyBId) |
|  |  | Fine-tuning scratch | [[101, 102]](https://www.zotero.org/google-docs/?vEguZ3) |
| MRI | Bones | Many | [[103]](https://www.zotero.org/google-docs/?vPiexR) |
|  | Genital systems | Feature extractor | [[30, 104]](https://www.zotero.org/google-docs/?rEjjOw) |
|  | Integumentary system | Fine-tuning scratch | [[105]](https://www.zotero.org/google-docs/?MXTD6x) |
|  |  | Many | [[106]](https://www.zotero.org/google-docs/?se4WJK) |
|  | Nervous system | Fine-tuning scratch | [[32, 107, 108]](https://www.zotero.org/google-docs/?fRk7Ag) |
|  |  | Many | [[35, 109–111]](https://www.zotero.org/google-docs/?mgLtzs) |
| OCT | Integumentary system | Feature extractor | [[112]](https://www.zotero.org/google-docs/?GZK7Jd) |
|  | Cardiovascular system | Many | [[113]](https://www.zotero.org/google-docs/?3WEQKb) |
|  | Sense organs | Feature extractor | [[114–117]](https://www.zotero.org/google-docs/?YGKeAZ) |
|  |  | Feature extractor hybrid | [[118]](https://www.zotero.org/google-docs/?XDGCmX) |
|  |  | Fine-tuning | [[41]](https://www.zotero.org/google-docs/?wB6CGK) |
|  |  | Fine-tuning scratch | [[49, 119]](https://www.zotero.org/google-docs/?wHBq1M) |
|  |  | Many | [[120–122]](https://www.zotero.org/google-docs/?xoArLY) |
| Photography | Integumentary system | Feature extractor | [[29, 123, 124]](https://www.zotero.org/google-docs/?SXjVhZ) |
|  |  | Fine-tuning | [[43]](https://www.zotero.org/google-docs/?JF41vy) |
|  |  | Fine-tuning scratch | [[125]](https://www.zotero.org/google-docs/?MwxL5c) |
|  | Else | Fine-tuning scratch | [[126]](https://www.zotero.org/google-docs/?RnAToT) |
| Sonography | Abdominopelvic cavity | Feature extractor | [[127]](https://www.zotero.org/google-docs/?52eZ1s) |
|  | Alimentary system | Feature extractor | [[128]](https://www.zotero.org/google-docs/?6kJocD) |
|  |  | Feature extractor hybrid | [[129]](https://www.zotero.org/google-docs/?iewd7C) |
|  |  | Fine-tuning scratch | [[130]](https://www.zotero.org/google-docs/?d8juLU) |
|  | Bones | Feature extractor | [[131]](https://www.zotero.org/google-docs/?wNK5NQ) |
|  | Endocrine glands | Fine-tuning scratch | [[132]](https://www.zotero.org/google-docs/?2XhxWh) |
|  | Genital systems | Feature extractor hybrid | [[133]](https://www.zotero.org/google-docs/?K4gf07) |
|  | Integumentary system | Many | [[134]](https://www.zotero.org/google-docs/?hnj1x1) |
|  | Respiratory system | Many | [[135]](https://www.zotero.org/google-docs/?ESvHJ6) |
|  | Urinary system | Feature extractor hybrid | [[136]](https://www.zotero.org/google-docs/?6Ce4wV) |
| SPECT | Nervous system | Feature extractor | [[137]](https://www.zotero.org/google-docs/?Pnbr9d) |
|  |  | Many | [[138]](https://www.zotero.org/google-docs/?s8G84I) |
| X-ray | Abdominopelvic cavity | Feature extractor | [[139]](https://www.zotero.org/google-docs/?pkd20v) |
|  |  | Feature extractor hybrid | [[140]](https://www.zotero.org/google-docs/?msSkn3) |
|  |  | Many | [[46]](https://www.zotero.org/google-docs/?iASEeT) |
|  | Alimentary system | Fine-tuning scratch | [[141–143]](https://www.zotero.org/google-docs/?3Xmu6m) |
|  | Bones | Feature extractor | [[144, 145]](https://www.zotero.org/google-docs/?5FfrZt) |
|  |  | Many | [[51, 146]](https://www.zotero.org/google-docs/?EM4C7i) |
|  | Cardiovascular system | Many | [[34]](https://www.zotero.org/google-docs/?9DCdwm) |
|  | Joints | Many | [[](https://www.zotero.org/google-docs/?ZC5GzI)14[7]](https://www.zotero.org/google-docs/?ZC5GzI) |
|  | Respiratory system | Feature extractor | [[28, 148–15](https://www.zotero.org/google-docs/?VL9Wth)0[]](https://www.zotero.org/google-docs/?VL9Wth) |
|  |  | Many | [[44, 15](https://www.zotero.org/google-docs/?87mYQI)1[]](https://www.zotero.org/google-docs/?87mYQI) |
|  | Thoracic cavity | Fine-tuning scratch | [[31]](https://www.zotero.org/google-docs/?scpJgm) |
|  |  | Many | [[52, 15](https://www.zotero.org/google-docs/?HYczY4)2[]](https://www.zotero.org/google-docs/?HYczY4) |
| Many | Many | Many | [[36, 15](https://www.zotero.org/google-docs/?0omJDV)3[]](https://www.zotero.org/google-docs/?0omJDV) |
