# Supplementary material for: Transfer learning for medical image classification: a literature review
Source: BMC Med Imaging. 2022 Apr 13;22:69. doi: 10.1186/s12880-022-00793-7 (PMC9007400; doi:10.1186/s12880-022-00793-7)
Supplement: Supplementary file 3 — Additional file 3. Summary table of public medical datasets. [file 12880_2022_793_MOESM3_ESM.docx]

# Appendix C. Supplementary public datasets

**Table 3** A summary table of public medical datasets. The abbreviations used on the task type field are as follows: C: Classification, D: Detection, R: Regression, Rg: Registration, S: Segmentation

| **Modality** | **Anatomical Part/Region** | **Task Type** | **Data** | **Published Year** | **URL** |
| --- | --- | --- | --- | --- | --- |
| CT scan | Abdomen | S | FLARE | 2021 | flare.grand-challenge.org |
|  |  | S | KiTS21 | 2021 | kits21.grand-challenge.org |
|  |  | S | SLIVER07 | 2019 | sliver07.grand-challenge.org |
|  | Cardiac | C | orcaScore | 2020 | orcascore.grand-challenge.org |
|  |  | S | CCTA | 2020 | asoca.grand-challenge.org |
|  | Head and neck | S | INSTANCE | 2022 | instance.grand-challenge.org |
|  |  | S | NucMM | 2020 | nucmm.grand-challenge.org |
|  |  | S | StructSeg | 2019 | structseg2019.grand-challenge.org |
|  |  | Many | CADA | 2020 | cada.grand-challenge.org |
|  | Spine | S | VerSe | 2020 | verse2020.grand-challenge.org |
|  | Thorax | C | STOIC | 2021 | stoic2021.grand-challenge.org/stoic-db |
|  |  | C | COVID19-CT | 2020 | covid-ct.grand-challenge.org |
|  |  | D | LUNA16 | 2016 | luna16.grand-challenge.org |
|  |  | R | LoDoPaB-CT | 2021 | lodopab.grand-challenge.org |
|  |  | Rg | EMPIRE10 | 2010 | empire10.grand-challenge.org |
|  |  | S | COVID-19-20 | 2020 | covid-segmentation.grand-challenge.org |
|  |  | S | LOLA11 | 2011 | lola11.grand-challenge.org |
|  |  | Many | RibFrac | 2020 | ribfrac.grand-challenge.org |
|  |  | Many | LNDb | 2020 | lndb.grand-challenge.org |
|  | Many | S | Parse | 2022 | parse2022.grand-challenge.org |
|  |  | Rg | CRC | 2018 | continuousregistration.grand-challenge.org |
| Endoscopy | Abdomen | D | EndoCV 2.0 | 2022 | endocv2022.grand-challenge.org |
|  | Pelvis | D | SARAS | 2021 | saras-mesad.grand-challenge.org |
| Microscopy | Tissues | C | BCNB | 2021 | bcnb.grand-challenge.org |
|  |  | C | HEROHE | 2020 | ecdp2020.grand-challenge.org |
|  |  | C | PatchCamelyon | 2019 | patchcamelyon.grand-challenge.org |
|  |  | D | MIDOG | 2021 | midog2021.grand-challenge.org |
|  |  | D | LYON | 2019 | lyon19.grand-challenge.org |
|  |  | S | WSSS4LAUD | 2021 | wsss4luad.grand-challenge.org |
|  |  | S | BCSS | 2021 | bcsegmentation.grand-challenge.org |
|  |  | S | SegPC | 2020 | segpc-2021.grand-challenge.org |
|  |  | S | PANDA | 2020 | panda.grand-challenge.org |
|  |  | R | BreastPathQ | 2019 | breastpathq.grand-challenge.org |
|  |  | R | LYSTO | 2019 | lysto.grand-challenge.org |
|  |  | Many | CoNIC | 2022 | conic-challenge.grand-challenge.org |
|  |  | Many | TIGER | 2021 | tiger.grand-challenge.org |
|  |  | Many | DigestPath | 2019 | digestpath2019.grand-challenge.org |
|  |  | Many | NuCLS | 2021 | nucls.grand-challenge.org |
|  |  | Many | PAIP | 2021 | paip2021.grand-challenge.org |
|  |  | Many | MoNuSAC | 2020 | monusac-2020.grand-challenge.org |
|  |  | Many | ACDC | 2019 | acdc-lunghp.grand-challenge.org |
|  |  | Rg | ANHIR | 2019 | anhir.grand-challenge.org |
|  |  | Many | ICIAR | 2018 | iciar2018-challenge.grand-challenge.org |
|  |  | Many | CAMELYON | 2017 | camelyon17.grand-challenge.org |
| MRI | Prostate | C | ProstateX | 2018 | prostatex.grand-challenge.org |
|  |  | S | PROMISE12 | 2012 | promise12.grand-challenge.org |
|  | Brain | Many | VALDO | 2021 | valdo.grand-challenge.org |
|  | Brain | S | FeTA | 2021 | feta.grand-challenge.org |
|  |  | S | BrainPTM | 2021 | brainptm-2021.grand-challenge.org |
|  |  | S | crossMoDa | 2021 | crossmoda.grand-challenge.org/CrossMoDA |
|  |  | S | Decathlon | 2018 | decathlon-10.grand-challenge.org |
|  | Knee | S | SKI10 | 2010 | ski10.grand-challenge.org |
| OCT | Eyes | C | ROCC | 2017 | rocc.grand-challenge.org |
|  |  | Many | AGE | 2019 | age.grand-challenge.org |
|  |  | Many | iChallenges | 2018 | ichallenges.grand-challenge.org |
|  |  | Many | RETOUCH | 2017 | retouch.grand-challenge.org |
| Photography | Eyes | C | AIROGS | 2022 | airogs.grand-challenge.org |
|  |  | C | RIADD | 2021 | riadd.grand-challenge.org |
|  |  | C | REFUGE | 2020 | refuge.grand-challenge.org |
|  |  | C | PALM | 2019 | palm.grand-challenge.org |
|  |  | C | ODIR | 2019 | odir2019.grand-challenge.org |
|  |  | C | ADAM | 2018 | amd.grand-challenge.org |
|  |  | C | IDRid | 2018 | idrid.grand-challenge.org |
|  |  | S | DRIVE | 2019 | drive.grand-challenge.org |
| Sonography | Breast | Many | ABUS | 2021 | tdsc-abus2023.grand-challenge.org |
|  | Brain | Many | CuRIOUS | 2019 | curious2019.grand-challenge.org |
|  | Fetal | R | HC18 | 2018 | hc18.grand-challenge.org |
|  |  | Many | A-AFMA | 2020 | a-afma.grand-challenge.org |
|  | Thyroid | Many | TN-SCUI | 2020 | tn-scui2020.grand-challenge.org |
| SPECT | Many | D | fastPET-LD | 2021 | fastpet-ld.grand-challenge.org |
| X-ray | Spine | R | AASCE | 2019 | aasce19.grand-challenge.org |
|  | Thorax | C | CXR-COVID19 | 2021 | cxr-covid19.grand-challenge.org |
|  |  | D | NOCE21 | 2021 | node21.grand-challenge.org |
| MRI; CT | Abdomen | S | CHAOS | 2021 | chaos.grand-challenge.org |
|  | Many | S | QUBIQ | 2021 | qubiq21.grand-challenge.org |
|  |  | Many | Learn2Reg | 2021 | learn2reg.grand-challenge.org |
| MRI; SPECT | Brain | Many | TADPOLE | 2017 | tadpole.grand-challenge.org |
| MRI; X-ray | Knee | C | KNOAP | 2021 | knoap2020.grand-challenge.org |
